# MicroRNA-34 and gastrointestinal cancers: a player with big functions

**DOI:** 10.1186/s12935-024-03338-w

**Published:** 2024-05-09

**Authors:** Wei Gao, Jianping Zhou, Mohammadamin Morshedi

**Affiliations:** 1 Department of Gastrointestinal and Hernia and Abdominal Wall Surgery, The First Hospital, China Medical University, Shenyang, 110001 China; 2 Research Center for Biochemistry and Nutrition in Metabolic Diseases, Institute for Basic Sciences, Kashan University of Medical Sciences, Kashan, Iran

**Keywords:** Gastrointestinal cancer, MicroRNA, miR-34, Exosome

## Abstract

It is commonly assumed that gastrointestinal cancer is the most common form of cancer across the globe and is the leading contributor to cancer-related death. The intricate mechanisms underlying the growth of GI cancers have been identified. It is worth mentioning that both non-coding RNAs (ncRNAs) and certain types of RNA, such as circular RNAs (circRNAs), long non-coding RNAs (lncRNAs), and microRNAs (miRNAs), can have considerable impact on the development of gastrointestinal (GI) cancers. As a tumour suppressor, in the group of short non-coding regulatory RNAs is miR-34a. miR-34a silences multiple proto-oncogenes at the post-transcriptional stage by targeting them, which inhibits all physiologically relevant cell proliferation pathways. However, it has been discovered that deregulation of miR-34a plays important roles in the growth of tumors and the development of cancer, including invasion, metastasis, and the tumor-associated epithelial-mesenchymal transition (EMT). Further understanding of miR-34a’s molecular pathways in cancer is also necessary for the development of precise diagnoses and effective treatments. We outlined the most recent research on miR-34a functions in GI cancers in this review. Additionally, we emphasize the significance of exosomal miR-34 in gastrointestinal cancers.

## Introduction

**Fig. 1:**
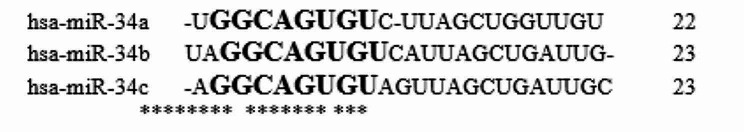
Sequence alignment of the mature miR-34a, miR-34b, and miR-34c. The seed-sequences are high-lighted in bold. CLUSTAL O(1.2.4) multiple sequence alignment

Cancer’s incidence and fatality rate have skyrocketed in recent years, making it one of the primary reasons for people’s passing away all around the globe [1]. Gastrointestinal (GI) cancers affects the GI systems, including the liver, rectum, colon, stomach, pancreas, and esophagus [2]. Both in industrialized and developing countries, gastrointestinal cancers are the most frequent form of the disease [3], In 2020, 3.5 million new instances of gastrointestinal cancer will be diagnosed, resulting in 2.2 million deaths worldwide [1]. Multiple GI malignancies are included in GLOBOCAN 2020’s 10 most common and deadly malignancies [1]. Approximately 10% of all cancer fatalities are a result of colorectal cancer. Colorectal cancer (CRC) is frequently seen in countries of the Western World. Meanwhile, both esophageal squamous cell carcinoma (ESCC) and gastric cancer (GC) are more common in East Asia; the combination of these two types of cancer is responsible for nearly 15% of all cancer fatalities worldwide. In younger individuals from industrialized nations, esophageal cancer and adenocarcinoma are on the rise, whereas rates of colorectal, pancreatic, and biliary cancer are stable or falling [1]. Some advanced gastrointestinal malignancies need systemic therapy, Molecular markers may improve patient outcomes by guiding therapy decisions [4–6]. MicroRNAs, often known as miRNAs, are short endogenous noncoding RNAs that range in length from 20 to 24 nucleotides that cause mRNA cleavage or impede coding for proteins by Watson-Crick base combining with target 3′-UTRs [7, 8]. MiRNAs govern 60% of the human protein-coding transcriptome, according to some research [9]. Aberrant expression of a single miRNA affects cellular pathways to cause cancer, differentiation, apoptosis, and survival signaling [7, 8]. Multiple studies have implicated miRNAs with carcinogenesis [10]. Studies have revealed that the members of the miR-34 family, miR-34c, miR-34b, and miR-34a, can prevent the forming of tumors and spur cell death in cancer cells. Figure 1 shows the sequence alignment of the mature miR-34a, miR-34b, and miR-34c. The seed-sequences are high-lighted in bold. Although the seed region in miR-34 family is exactly the same, but because the majority of the reviewed sources are related to miR-34a, the focus of this study is on this miRNA. The important thing is that by checking the tissueatlas2 database, we will find that the expression of miR-34a is much higher in our target tissues compared to the other two members of this family, and in other words, these two members can be ignored and the observed effects related to has-miR-34a-5p (miR-34a) (https://ccb-web.cs.uni-saarland.de/tissueatlas2/patterns/hsa/mirna/hsa-miR-34b-5p/; https://ccb-web.cs.uni-saarland.de/tissueatlas2/patterns/hsa/mirna/hsa-miR-34c-5p/; https://ccb-web.cs.uni-saarland.de/tissueatlas2/patterns/hsa/mirna/hsa-miR-34a-5p/ ).

Certain gene sequences are chosen by the microRNAs, specifically those associated with the cell cycle (CDK4, CDK6, and CCND1) and those meant to stop cells from dying (BCL2, SIRT1). This selection is intentional and is to serve the functions of the microRNAs. That’s why they’re so important as tumor-suppressing miRNAs [11]. Tumor suppressor miRNAs play a crucial role in preventing the initiation of cancer by regulating the expression of genes that code for oncoproteins [12, 13]. Numerous research studies have indicated a correlation between the decrease in certain miRNAs and the advancement of cancer. Yet, only specific tumor suppressor miRNAs that are frequently diminished in cancerous cells are responsible for causing a strong effect. This phenomenon has been observed in multiple miRNA families that have gained significant recognition, such as the well-known tumor-suppressing let-7, miR-15/16, and miR-200. These miRNAs act as suppressors of tumors by silencing their targeted oncogenic mRNA network, ultimately hindering the process of tumorigenesis. MiR-34 relatives (miR-34a, miR-34b/c) are spread out over two different chromosomal locations [11], This suggests they may be controlled by somewhat distinct transcriptional and epigenetic processes. P53 directly regulates miR-34a, and CpG methylation deactivate it in many malignancies [14, 15]. . One kind of miRNA is called microRNA-34a (miR-34a) that is shown get aberrant expression in many types of cancer, including gastric cancer (GC) [16], esophageal cancer (EC) [17], hepatocellular carcinoma (HCC) [18], colorectal cancer (CRC) [19], pancreatic cancer (PC) [20], gallbladder cancer (GBC) [21], and others [22]. Fresh research suggests a link between miR-34a and the development of gastrointestinal cancer [23], invasion [24] and metastasis [25], showing that miR-34a plays crucial physiologic functions in cellular signaling networks such MAPK/Ras [26], Wnt/-Catenin [27], PI3K/Akt [28], SIRT1/p53 [29], and FoxM1/c-Myc pathway [30].

Some classes of miRNAs function as Cancer-fighting genes also may block carcinogenesis by Reduce oncogenes, suggesting that miRNAs is promising for potential use in cancer therapy as a diverse disease [31]. miRNAs have a synergistic therapeutic impact in cancer because they may target numerous oncogenes or biological pathways all at once [32]. Additionally, in comparison to plasmid DNA-based gene therapy and protein-based pharmaceuticals, miRNAs as exogenous antisense nucleotides shown much less immunotoxicity [33]. Accordingly, Cancer therapy may benefit greatly from the study of microRNAs. It’s well-known that miRNAs are known to affect prognosis, diagnosis, therapy, and medication resistance of GI cancer [34–39]. We outlined the most recent research on miR-34a functions in GI cancers in this review. Additionally, we emphasize the significance of exosomal miR-34 in gastrointestinal cancers.

## MircroRNA-34 and pancreatic cancer

**Fig. 2:**
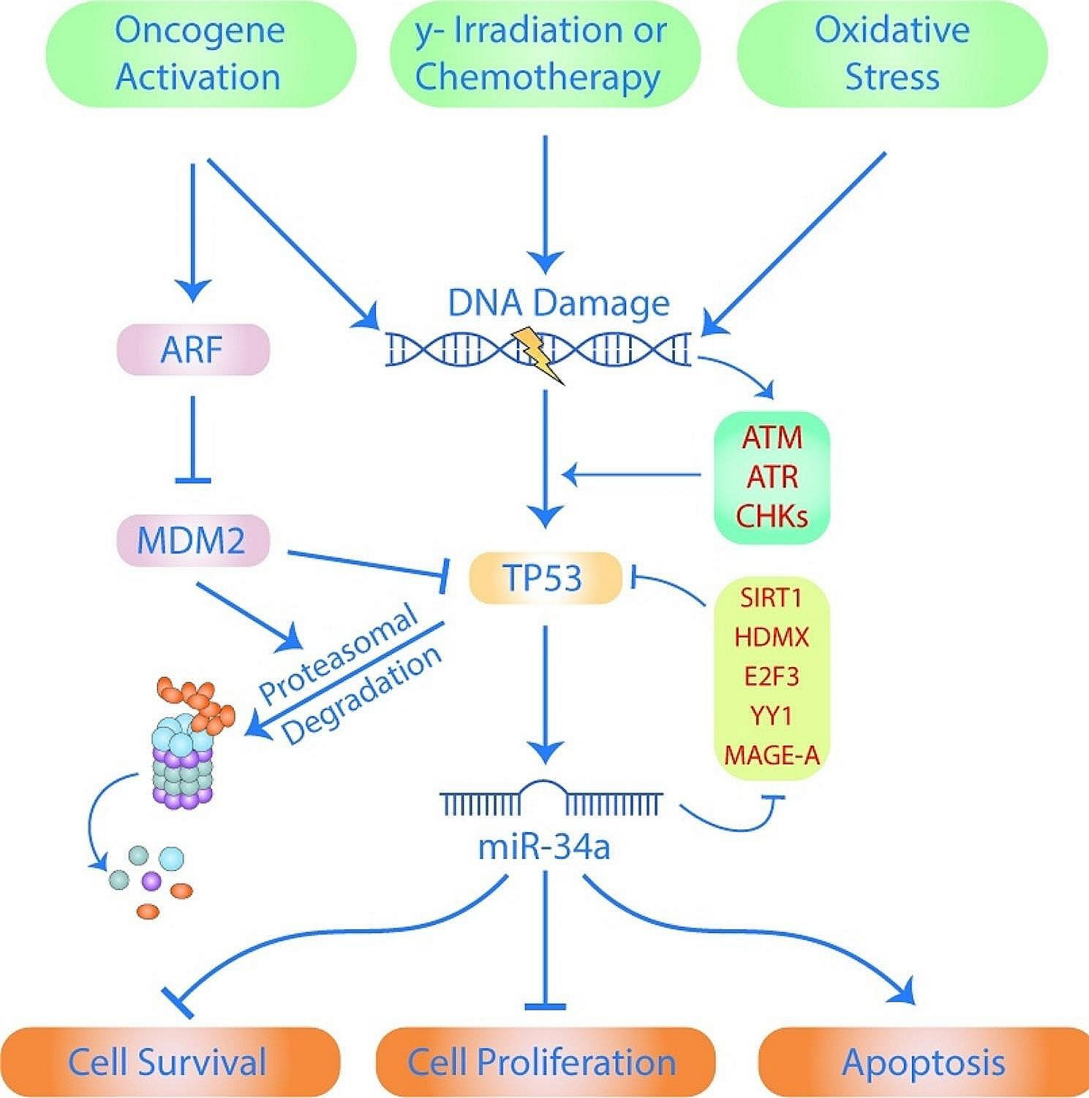
The cycle of interactions involving the TP53 gene that regulates miR-34a levels. This figure adapted from [79]

Pancreatic ductal adenocarcinoma (PDAC) has been revealed to contain approximately 70 distinct miRNAs that are aberrantly expressed, influencing metastasis, proliferation, EMT, metabolism, and apoptosis. Tp53 gene dysfunction and an active mutation of the oncogene Kras are the most major genetic changes driving PDAC. Mir34a may be of utmost importance in the control of pancreatic ductal adenocarcinoma (PDAC) as it is specifically targeted by the Tp53 protein (Fig. 2) [40]. 90% of pancreatic ductal adenocarcinoma (PDAC) cases are caused by a mutation in the KRAS oncogene that turns the gene permanently on [41]; G12D substitution accounts for 41%, G12V for 34%, and G12R for 16% of these occurrences, respectively [42]. When KrasG12D is expressed in the epithelium, paracrine signaling is initiated. All lesion types that are generated by the normal exocrine pancreas, including pancreatic ductal adenocarcinoma, ductal metaplasia, and pancreatic intraepithelial neoplasia, are due to a fibro-inflammatory setting induced by desmoplasia associated with pancreatic ductal adenocarcinoma [43]. Progression of the illness is also fueled by the inability of tumor suppressor genes including p53, p16, and SMAD4 to operate [44].

An in vivo analysis of Mir34a’s tumor suppressive effect in PDAC utilizing genetically altered mice models is presented by Hidalgo-Sastre et al. [45]. Mice with a mutation in the oncogene Kras (KrasG12D; Mir34a/) in the pancreas were bred to test the idea that Mir34a performs inhibitor of PDAC development in vivo. Compared to KrasG12D controls, KrasG12D; Mir34a/ mice developed PDAC more quickly and had more advanced pre-neoplastic lesions, as shown by histological investigation. An investigation revealed that the increased expression of the pro-inflammatory cytokines TNFA and IL6 in regular acinar cells stimulates the accelerated phenotype, possibly caused by the migration of immune cells. Their research indicates that Mir34a suppresses PDAC growth through controlling the immunological environment of the tumor, suggesting that reactivating Mir34a could be a helpful therapeutic strategy for slowing the disease’s progression [45].

The location of the SERPINE1 gene, which codes for a 50 kDa single-chain glycoprotein, has been identified at 7q21.2-q22. One of the significant parts of the plasminogen activator system is the SERPINE1 gene (PAs). This gene is responsible for delaying the activation of plasminogen into the protease enzyme plasmin by suppressing the action of tissue-type plasminogen activator (tPA) and urokinase-type plasminogen activator (uPA) [46]. The SERPINE1 gene encodes Plasminogen activator inhibitor-1 (PAI-1), which contributes to the control of the migration and the penetration of tumor cells [47, 48]. There are two possible states for the PAI-1 protein: an active and a dormant one. The conformation of PAI-1 is significant because it activates Multiple cell signaling pathways contribute to malignant progression [49]. SERPINE1 has been identified as a miR-34a target in colorectal [50] and non-small cell lung cancer by prior studies [51].

To identify WT-primary TP53’s targets in PDAC, Akula et al. investigated the expression in MIA-PaCa-2 cells of 440 PDAC-derived proteins [49]. Due to the TP53 and KRAS mutations in MIA-PaCa-2 cells, PDAC can be effectively researched in vitro utilizing these cells. In cells lacking wild-type TP53, RPPA detected the synthesis of tumor-promoting proteins. By utilizing a vector to express Wild-Type TP53 (WT-TP53) or through administration of berberine or a modified form of berberine (BBR), these impaired states were significantly ameliorated. When compared to cells expressing mutated TP53 (mTP53), the cells expressing WT-TP53 had higher expression of the miR-34a-associated signaling. Their in vivo research, examining human PDAC specimens, miR-34a and related signaling was reduced considerably in the pancreatic cancer tissues compared to the non-cancerous specimens, which is consistent with the findings from their in vitro analysis. Their research also indicated that SERPINE1 is a miR-34a target that plays a crucial role in PDAC biology. By uncovering the key target (TP53/miR-34a axis), they created a useful biomarker (SERPINE1) to aid in the early detection of pancreatic cancer [49].

Previous investigations have revealed a relationship between Notch signaling and the epithelial-mesenchymal transition (EMT) and its relationship to the origination and evolution of pancreatic cancer [52–54]. EMT is a program that can be reversed and is characterized by a process wherein epithelial cells become mesenchymal cells [55]. In addition to its involvement in tissue regeneration, cancer metastasis and embryonic development all rely on EMT. E-cadherin protein is lost during the EMT program, and N-cadherin, vimentin, and fibronectin are obtained as mesenchymal markers instead [52]. Several signaling pathways, including as Notch and TGF-, contribute to EMT (transforming growth factor-). Recent research has revealed that Notch signaling and EMT share a number of overlapping roles in the control of cell viability [56, 57]. Snail and ZEB, transcription factors that down regulate E-cadherin and upregulate N-cadherin, respectively, are involved in TGF-mediated EMT [58, 59]. EMT and Notch signaling regulate pancreatic cancer therapy.

Tang et al. wanted to know if miR-34a increases the risk of pancreatic cancer and whether this increase is linked to Notch signaling and EMT [60]. Pancreatic cancer cells’ invasion and migration were inhibited by miR-34a in vitro, but were increased by miR-34a inhibitors (PANC-1 and SW-1990). These effects could be overcome by Snail1 overexpression or shRNA. Notch1 shRNA decreased MiR-34a’s anti-apoptotic effects on pancreatic cancer cells. MiR-34a targets Snail1 and Notch1, according to luciferase reporter assays. MiR-34a inhibited pancreatic cancer growth in vivo by reducing the expression of Snail1 and Notch1. The expression of Snail1 and Notch1 is regulated by MiR-34a, which inhibits the spread of pancreatic cancer [60].

For the X chromosome to become inactive in female mammals, an 18 kb gene-silencing long non-coding RNA called X inactive-specific transcript (XIST) is required [61]. LncRNAs and miRNAs interact in many forms of cancer, according to research [62–65], and so add additional mysteries to the regulatory networks of miRNAs and lncRNAs.

Sun et al. looked into how pancreatic cancer (PC) growth and progression are impacted by the XIST [66]. They first noticed that PC tissues and PC cell lines both had much higher levels of the lncRNA XIST. PANC-1 cells’ migration, invasion, and proliferation were significantly reduced when XIST was knocked down, and their rate of apoptosis was accelerated. In contrast, BxPC-3 cells’ expression of XIST was significantly boosted. BxPC-3 and PANC-1 cells were subcutaneously injected into nude mice to assess tumor development. The cells had been transfected with various vectors. Studies conducted in vivo proved that XIST promoted tumor growth. Another research revealed that low miR-34a-5p (miR34a-5p) levels in PC tissues were indicative of a poor prognosis. miR-34a-5p was also demonstrated highly negatively linked with XIST, indicating that it might be an XIST target gene. Finally, they discovered that miR-34a-5p counteracted XIST’s ability to promote malignant behavior. XIST and miR-34a-5p were identified as possible therapeutic targets for PC based on these findings [66].

miRNAs may negatively impact critical genes that are involved in stem cell self-renewal and survival, according to mounting evidence [67–70]. Recent research has shown that the three miR-34 family members are directly regulated by p53, and miR-34’s functional activity has been linked to tumor-suppressive qualities [14, 71–75]. miR-34 has been observed to have direct control on the Bcl-2 protein [76]. The results of He and his colleagues showed that as a result of creating abnormal levels of miR-34, cell cycle progression was halted in cells from both healthy and cancerous tissue, which confirms previous research that miR-34 is capable of reducing the activity of genes that move the cell cycle forward [14]. According to a study, MiR-34 expression was markedly down regulated in p53-mutant gastric cancer cells, and boosting miR-34 expression prevented the cancer cells from proliferating [72]. . MiR-34 loss causes cancer chemoresistance [77]. miR-34a is implicated in colon and pancreatic cancer p53-mediated apoptosis [73, 74].

Ji et al. looked into the connection between miR-34 and pancreatic cancer stem cells by investigating the part miR-34 plays in the human pancreas cancer cell lines MiaPaCa2 and BxPC3 which have mutated p53 [78]. Upon reintroduction of miR-34 into pancreatic cancer cells through either using miR-34 mimics to transfect or through lentiviral MiR-34-MIF infection, a decrease of Bcl-2 and Notch1/2 occurred. This subsequently led to restricted G1 and G2/M cell cycle, increased susceptibility to chemotherapy, and decreased growth and invasion of clonogenic cancer cells. In CD44+/CD133 + MiaPaCa2 cells, a decrease in miR-34 presence was combined with a rise in Notch/Bcl-2 and a higher concentration of tumor stem/progenitor cells, tumor sphere-forming cells, and tumor initiating cells. MiR-34 restoration greatly reduced both the formation of tumorspheres in vitro and the growth of tumors in vivo, as well as the amount of tumor-initiating cells (by 87%). Our findings suggest that miR-34 may partially restore the tumor-suppressive properties of p53-deficient human pancreatic cancer cells. Their findings are consistent with the theory that miR-34 controls the capacity of pancreatic cancer stem cells to self-renew and/or decide their own fate, presumably by directly influencing their targets Bcl-2 and Notch. P53-miR34-deficient human pancreatic cancer may benefit greatly from miR-34 recovery as a new molecular therapeutic, presumably through suppressing pancreatic cancer stem cells [78]. Table 1 presents information detailing various research conducted on the topic of miR-34 and its relation to pancreatic cancer.

**Table 1:** Various studies on miR-34 and pancreatic cancer

Type of miR-34	Expression	Target (s)	Type of model	Tumor cell line	Citation
miR-34	Down	Bcl-2, Notch	In vitro, in vivo	MiaPaCa2, BxPC3, WI-38	[78]
miR-34a	Down	SMAD4, IL-6R, Notch1	In vivo	-	[45]
miR-34a	Down	Snail1, Notch1	In vitro In vivo	PANC-1, SW-1990, HPDEC	[60]
miR-34a	Up	NAV3, ACSL1, AKAP6, CAPN6, CORO1C, CTNND2, E2F5, EML5, JAG1, KIAA1217, LEF1, LGR4, MAP2K1, NOTCH1, PDGFRA, PNOC, TMEM55A, UHRF2	In vitro	AsPC-1, CAPAN-2, PC-1.0, PC-1	[80]
miR-34a	Down	Notch pathway	In vivo In vitro human	HPC-Y5, HPDE6-C7, PANC-1, PC PDCs, Capan-1, PDCs No. 2, and No. 4, PDCs No. 1, and No. 3, HCC cells	[81]
miR-34a	Down	c-myc, Bcl-2	In vitro	MIA PaCa-2, Capan-1, HPDE	[55]
miR-34a	Down	XIST	In vitro In vivo human	PANC-1, ASPC-1, MIA PaCa-2, HPAC, CFAPC-1, BxPC-3, HPDE, 293T cells	[66]
miR-34a	down	SNAI1 SNAI2	In vitro	A549, LC-2/ad, Panc1	[82]
miR-34a	Down		In vitro In vivo	BxPC3, CAPAN2, CFPAC1, PANC04.03, PANC1, SW1990, HEK293T,	[83]
miR-34a	Down	Bcl-2, CDK6, SIRT1, Notch pathway,	In vitro	MIA PaCa-2, AsPC-1, CSCs	[84]
miR-34a	Down	SERPINE1, ATG4B, AXL, GATA3, JAG1, LDHA, MAP2K1, MYT1, NOTCH1, PEA-15, SERPINE1, SNAIL, PCD4, MAPT	In vitro	MIA-PaCa-2,	[85]
miR-34a	Down	-	In vitro	HCT116	[74]
miR-34a	Down	XIST	In vitro human	AsPC-1, MIAPaCa-2, PATU8988T, PANC-1, SW1990, HPDE6-C7, HEK-293T	[86]
miR-34a	Down	SIRT1	In vitro In vivo	MiaPaCa-2	[87]
miR-34c	Down	ABCC3	In vitro In vivo	AsPC1, HPAFII, CFPAC-1, BxPC-3, Capan-1, Capan-2, hTERT-HPNE, PZR1, PZPR1, PZPflR, pcDNA3-ABCC3	[88]

## MircroRNA-34 and gastric cancer

The TP53 gene on chromosome 17p13.1 encodes the 393 amino - acids long nuclear p53 protein, which is essential for maintaining genome integrity [89]. Research has demonstrated that the transcription factor known as p53 has a role in the apoptotic process by targeting many components [90]. New research has shown that p53 contributes to carcinogenesis by modulating miRNAs in yet another mechanism [91–93]. Corney et al. (2007) found miR-34c and miR-34b jointly target p53, purposely decrease cell division and development without adhesion. Expression of miR-34b/c was also upregulated because p53 binds to its promoter In humans, a non-synonymous change at codon 72 of the p53 protein causes a proline to be replaced by arginine (Arg72Pro, rs1042522) [94]. The biological features of the Arg72Pro polymorphism are different, with the 72Pro variation displaying a lower risk for apoptosis than the 72Arg variant [95]. In light of this, research shows looked at the connection among the Arg72Pro polymorphism also different forms of malignancy, including gastric cancer [96–98]. The promoter of pri-miR-34b/c contains the polymorphism rs4938723T/C [99]. It has been demonstrated that GATA-X binding is impacted by changing the rs4938723 polymorphism from T to C and, in turn, promoter transcription activity [99–101]. Hepatocellular carcinoma is only one of several cancers linked to the polymorphism rs4938723 in miR-34b/c, which has generated a lot of research [99, 102, 103], colorectal cancer [103, 104], nasopharyngeal carcinoma [104], esophageal squamous cell carcinoma [105, 106], osteosarcoma [106] and renal cell cancer [107].

Pan et al. looked at the connection between a polymorphism in TP53 (Arg72Pro) and a variant in miR-34b/c (rs4938723), both of which connected to a higher risk of GC [108]. They examined the frequency of such two polymorphisms in a population of 289 controls and 197 GC patients who shared the same gender, age, geographic location, and ethnicity. They carried out this utilizing DNA direct sequencing, length of restriction fragment polymorphism, and the polymerase chain reaction. miR-34b/c rs4938723 genotype CT or CT/CC individuals had significantly lower odds of developing GC than TT individuals (OR = 0.66; 95% CI, 0.45–0.97; and OR = 0.67; 0.47–0.97). It was discovered that those possessing the miR-34b/c rs4938723 CT/CC and TP53 CG/CC components displayed a 0.62-fold lesser chance to become afflicted by stomach cancer in comparison to people with miR-34b/c rs4938723 TT and TP53 CG/CC genotypes, implying that miR-34b/c rs4938723 could possibly stop the onset of gastric cancer [108].

IGF2BP3, also known as IMP3, a protein that bind mRNA for insulin-like growth factor 2. The elevated levels of IGF2 binding protein 3 in pancreatic cancer led to its discovery [109]. Soon after its discovery, IGF2BP3’s role as an overexpressed family member in tumors like squamous cell carcinoma was clarified [110], lung cancer [111], melanoma [112], colon cancer [113], liver cancer [114]. This may have an oncogenic function in cancer, since its expression was abnormally upregulated. Additionally, growing data suggested that IGF2BP3 was a useful biomarker for several malignancies, including colon cancer [115] and GC [116].

Zhou et al.; studied IGF2BP2’s function in stomach cancer [117]. They employed real-time polymerase chain reaction (qRT-PCR) and Western blotting to analyze IGF2BP3’s expression in both GC cell lines and original tissues, and they utilized a series of in vitro functional experiments to measure IGF2BP3’s biological activity. Using luciferase assays and rescue studies, TargetScan predicted that it was regulated by miRNAs, and those predictions were verified. After doing an expression microarray study on GC cell lines, IGF2BP3 was shown up-regulated gene. IGF2BP3 expressed more in GC tissues than in normal gastric epithelium. Poor disease-specific survival was associated with increased IGF2BP3 expression. Removal of IGF2BP3 dramatically reduced cell invasion and proliferation. IGF2BP3 is known to be suppressed by tumor-suppressive miRNA, particularly miR-34a, in addition to copy number growth. Investigators found that primary gastric cancer (GC) samples had a negative correlation between IGF2BP3 mRNA expression and miR-34a expression. Augmenting expression of IGF2BP3 nullified the inhibitory consequences of miR-34a. They briefly explained its carcinogenic importance and demonstrated with certainty how the silencing of miR-34a leads to the activation of IGF2BP3 in gastric carcinogenesis. Their research uncovered a promising predictive biomarker with use in the clinic [118]. .

Chemotherapy is a key method for treating gastric cancer, however it is undermined by multidrug resistance (MDR) GC [119]. In a wide range of malignancies, miRNA abnormalities are crucial for the emergence and maintenance of MDR. The evolution of GC and MDR has been linked to the histone deacetylase Sirtuin 1 (SIRT1) [120, 121]. SIRT1 may aid in the creation of MDR by regulating the amount of specific proteins, including P-gp and MRP1, responsible for resistance [122, 123]. At SCG-7901 cells, miR-34a was found to decrease SIRT1 expression [124]. Moreover, a variety of malignancies’ start and progression are significantly influenced by hsa-miR-34a-5p [125–127], and chemoresistance influence them too [127–129].

Deng et al. conducted an examination of the expression levels and actions of hsa-miR-34a-5p and SIRT1 to better understand how they work together to modulate the MDR and reaction to chemotherapy of GC cells [130]. Results of studies on the effects of SGC-7901/5-Fu on multi-drug resistant GC cells showed that the concentration of hsa-miR-34a-5p was less in comparison to normal SGC-7901 cells. When the cells were exposed to chemotherapy, there was an increased expression of hsa-miR-34a-5p which caused apoptosis, and consequently prohibited the migration and invasion of drug-resistant GC cells. The amplified hsa-miR-34a-5p was also found to impede the growth of a drug-resistant tumor in vivo. Speaking to how hsa-miR-34a-5p achieves its purpose, it is suspected that it binds with the target genes contained in the 3’-untranslated region (UTR). In the course of further investigations, target genes involved in the phenomenon, such as MRP1, P-glycoprotein, and SIRT1, were identified. It was inferred that hsa-miR-34a-5p inhibited SIRT1, P-gp, and MRP1, thus making multi-drug resistant GC cells more susceptible to chemotherapy. Additionally, it was noted that the overexpression of hsa-miR-34a-5p may mitigate the resistance of GC cells to multiple drugs by decreasing the expression of SIRT1, P-gp, or MRP1 [130].

Tgif2 has been associated to numerous cancer types. Many ovarian cancers owe their genesis or advancement in part to TGIF2 over-expression [131]. MiR-34a might contribute controlling its expression. MiR-34a has been shown in a study to decrease osteoclastogenesis and Tgif2, hence preventing bone metastases and osteoporosis [132].

Hu et al. conducted a study to evaluate if the expression levels of Tgif2 and miR-34a have an effect on gastric cancer (GC) tissues and adjacent normal tissues. To do this, western blotting and real-time polymerase chain reaction (PCR) methods were applied. On top of this, Tgif2 siRNA or miR-34a mimic was administered to the tissues and the expression levels were tested again using western blotting and PCR. The water-soluble tetrazolium salt and an Annexin-V-FITC Apoptosis Detection Kit I were utilized to observe cell proliferation and apoptosis, respectively. The results showed that the expression of miR-34a was higher in normal tissues compared to malignant tissues, whereas the expression of Tgif2 was the opposite. When transfected with the miR-34a mimic and Tgif2 siRNA, the Tgif2 expression decreased significantly in the GC cells. It was also seen that the cell proliferation was lower and rate of apoptosis was higher in the cells exposed to both the miR-34a mimic and Tgif2 siRNA contrastive to those not exposed to them. Overall, these findings show that miR-34a may be a potential therapeutic tool for controlling tumor invasion and metastasis due to its ability to suppress Tgif2 expression in GC [133].

A protein called survivin, also referred to as baculoviral suppressor of apoptosis repeat-containing 5 (BIRC5), triggers apoptosis to prevent cells from deteriorating (IAP). Upregulation of survivin occurs in almost every human tumor because of its role in and cell cycle control [134, 135]. GC patients who had an abnormally high level of survivin expression were thought to have a better chance of survival [136, 137].

In their research, Yang et al. examined the link between perseverance of survivin and the propagation and infiltration of gastric cancer cells affected by miR-335 and miR-34a and identified a connection between the amount of miR-335 and/or miR-34a expressed and the overall survival of those afflicted with GC [138]. A trial was conducted to observe the following fifty GC patients that had been initially diagnosed between 2011 and 2015. Utilizing miRNA microarray technology, the miRNA expression pattern was investigated in eight tumors and the surrounding healthy cells. Additionally, qRT-PCR, Western blotting, and a luciferase assay were used to validate the assumed link between miR-335 and survivin mRNA. Researchers later conducted studies to understand the role of miR-335 and miR-34a on gastric cancer cell’s proliferation, apoptosis, and invasion. This was done through Transwell assay, flow cytometry, and the CCK-8 test. Furthermore, the latency between decreased miR-335 or miR-34a expression and lymph node metastasis in larger tumors was also investigated. Results showed that if miR-335 or miR-34a was absent, the prognosis was poor; and if both miRs were absent the prognosis was even worse. miR-335 works directly to suppress the synthesis of survivin by connecting to the 3’-UTR region, whereas miR-34a acts more indirectly and functions to restrict cell invasion and proliferation in addition to promoting apoptosis. However, increased expression of survivin that did not have the 3’-untranslated region was able to counteract these functions. Overall, our findings demonstrate the potential influence miR-335 and miR-34a have on restraining GC cell proliferation, apoptosis, and invasion despite previous theories [138]. Table 2 presents details of multiple investigations concerning the role of miR-34 in cases of gastric cancer.

**Table 2:** Various studies on miR-34 and gastric cancer

Type of miR-34	Expression	Target (s)	Type of model	Tumor cell line	Citation
miR-34	----	G1/S cell cycle checkpoint regulation pathway	human	-	[139]
miR-34	down	Notch, HMGA2, Bcl-2, Bax	In vitro	Kato III, AGS, N87, MKN45, WI-38	[92]
miR-34	down	YY1	In vitro In vivo	SC-M1, AGS, AZ521, NUGC-3, KATO III, NCI-N87, SNU-16	[140]
miR-34b/c	down	p53	human	-	[108]
miR-34	down	-	In vivo In vitro human	SNU-601, SNU-638, AGS	[141]
miR-34	Up	-	In vivo	-	[142]
miR-34a	Up	-	human	-	[143]
miR-34a	down	LDHA	In vitro human	human gastric epithelial cell line, gastric cancer cell lines	[144]
miR-34a	Up	-	In vitro	SGC-7901 (BNCC100674)	[145]
hsa-miR-34a-5p	down	SIRT1	In vivo In vitro	SGC-7901	[130]
miR-34a	Up	-	In vitro human	MKN-45, SGC-7901, N87, GES-1	[146]
miR-34a	down	Survivin, BIRC5	In vivo In vitro	NCI-N87, MKN-28, AGS, HGC-27, SGC-7901, GES-1	[118]
miR-34a	Up	-	In vivo In vitro	AGS, BGC-823	[147]
miR-34a	down	Notch-1 signaling pathway	In vivo In vitro	BGC-823, GES-1	[148]
miR-34a	down	-	In vivo In vitro human	GES-1, HFE145, BGC823, SNU-719, MGC803, AGS, MKN-45 MKN-28	[149]
miR-34a	down	SIRT1	In vitro human	GES-1, AGS, SGC‑7901, MKN‑45, BGC‑823 and 293	[124]
miR-34a	down	Tgif2	In vitro	GES-1	[133]
miR-34a	down	HK1, p53, MAPK	In vivo In vitro human	AGS, BGC823, SGC7901	[150]
miR-34a	down	Notch1, β-catenin	In vitro	AGS, KatoIII, BEAS-2B	[151]
miR‑34a	down	β-catenin, Notch1	human	-	[152]
miR‑34a	down	PI3K	In vitro	SGC-7901, SGC-7901/DDP	[153]
miR‑34a	down	c-Met	In vitro human	SGC7901 GC, normal gastric epithelial cells	[154]
miR‑34a	down	-	In vitro human	GES‑1, NCI‑N87, AGS, MKN‑45, MKN‑28, BGC‑823, SGC7901	[155]
miR‑34a	Up	-	In vitro	Chrysin, PEG(6000), MTT, Sn(Oct)2 ,DCM, DMSO, PVA, D,LLactide AGS	[156]
miR‑34a	Down	PI3K, Wnt	In vivo In vitro human	SGC7901 ,MGC803, GES1	[157]
miR‑34a	down	Bcl-2, c-myc, E2F3	In vitro human	BGC-823, SGC-7901	[16]
miR‑34a	down	IGF2BP3	In vivo In vitro	MKN1, MKN7, MKN28, MKN45, SNU1, SNU16, AGS, KatoIII, NCI-N87, MGC-803, SGC-7901, TMK-1, GES-1, HFE-145	[117]
miR‑34a	Up	DAPK2, Sp1, E2F-1, caspase3, caspase7	In vivo In vitro	MGC-803, SGC7901/DDP	[158]
miR‑34a	down	Survivin	In vitro	MGC80-3, SGC-7901, HGC-27, NCI-N87, GES-1	[159]
miR‑34a	down	PDGFR, MET, E2F3, PI3K	Human In vitro	HEK 293 T, AGS, GES-1, BGC-823, MGC-803, SGC-7901	[138]
miR‑34a	down	-	Human	-	[160]
miR‑34a	down	LDHA	Human In vitro	Jurkat cell line	[161]
miR‑34a	down	PDGFR, MET, PI3K	Human In vitro	SGC-7901, HGC-27, AGS, MKN-45, N87, GES-1	[28]
miR‑34a	down	MET,	Human In vitro	SGC7901, SGC7901/DDP,	[136]
miR‑34a	Up	CD44	In vitro	SGC-7901, MGC-803,	[137]
miR‑34a	down	PI3K	Human In vitro	MKN-45, AGS,	[162]
miR‑34a	down	-	Human In vitro	AGS, MKN1	[25]
miR‑34a	up	E2F3; CCND1; CDK6	In vitro	MKN-45	[163]
miR‑34a	down	EMT-inducing Notch, Wnt, TGF-β1, NF-κB signaling pathways	human	-	[164]
miR-34a	up	TNF, TNFR1 ,TNFR2 ,TRADD, TRAF2 ,CFLIP, CASP8 ,CASP3 ,NFKB1	human	-	[165]
miR-34a	down	-	Human In vitro In vivo	MTT	[166]
miR-34a	down	PI3K	human In vitro	SGC‑7901	[167]
miR-34a	down	CD44, NANOG	In vitro In vivo	MKN-74	[168]
miR-34a-5p	up	-	Human	-	[169]

## MircroRNA-34 and colon cancer

Integral membrane protein SYT1 (synaptotagmin I) on 12q21.2 had a significant impact in synovial sarcomas. SYT1 is a candidate ring chromosome and has structural rearrangements with three separate genes [170]. Utilizing a 32 K BAC array, Nord et al. identified SYT1 as a new oncogene in glioblastoma [171]. Important fresh visions into the molecular genetic basis of colon cancer were offered by Zhu H et al., who also found that SYT1 might be used as a potential marker in the disease [172].

Hichao et al. utilized qRT-PCR to analyze the miR-34a expression in both colon cancer tissues and associated cell lines [173]. An examination was conducted to evaluate the function of miR-34a and SYT1 in the proliferation, invasion, migration, and apoptosis of cells through in vitro cell functional studies. A fluorescent protein-labeled reporter assay revealed an interaction between miR-34a and SYT1; when the levels of SYT1 were raised, the effects of miR-34a on cell growth and death were reversed, thereby confirming that the two had a causative connection. Further analysis of human colon cancer specimens uncovered that miR-34a was expressed in smaller amounts than in healthy colon mucosal epithelial cells; in addition, miR-34a upregulation hampered cell proliferation, migration, and invasion while down regulation caused cell mortality in vitro. Evidence that miR-34a directly targets the 3’ untranslated region (UTR) of SYT1 suggested that miR-34a affected SYT1 in colon cancer. When SYT1 was deactivated, execution of cell migration, invasion, and proliferation was severely restricted, along with raised cell death, indicating that SYT1 possibly serves as an oncogene in colon cancer. A significant decrease in cell proliferation and an increase in cell death were both observed when SYT1 was increased in the absence of miR-34a. Altogether, these conventions signify that miR-34a and SYT1 are essential elements for the proliferation of colon cancer [173].

The original tumor cells undergo a process known as EMT which is responsible for the further development of metastasis [174]. The production of EMT is stimulated by multiple signaling molecules which are released by tumor cells. These cytokines are able to activate receptor tyrosine kinases (RTKs) [175]. The class of RTKs which is inclusive of the PDGFR and c-Kit receptors extends to include the CSF1R, which is coded by the c-fms gene [176–178]. Both c-fms and v-fms have been identified as having the power to transform [179, 180]. The binding of CSF1 to its latest ligand, interleukin 34 (IL34), causes the homodimerization of CSF1R and brings about its activation [181, 182]. Subsequently, associated successive signaling pathways such as PI3K/AKT, JAK/STAT, and Ras/Raf/MAPK are initiated [182–184]. Targeting CSF1R in TAMs is a viable method for treating these cells since they rely on this receptor to carry out essential processes [185]. Little is known, however, about the importance of CSF1R expression in tumor cells with an epithelial background. It is noteworthy that breast cancer metastasis and progression are associated with increased expression of CSF1 and CSF1R [186]. Patients with advanced colorectal cancer have considerably increased serum CSF1 levels, which may indicate a function for CSF1R signaling in the growth of CRC [187].

Shi et al. determined that the microRNA miR-34a has a direct impact on the tyrosine kinase receptor CSF1R, but also serves as the initiator of p53 and miR-34a working together to restrict colorectal cancer [188]. The investigators developed a plan to anticipate death and group by molecular patterns in the TCGA-COAD and other three colorectal cancer cohorts using mRNA expression data. They identified and experimentally ratified targets for microRNAs and transcription factors, examining the workings that influence acquired chemoresistance, metastasis, migration, and EMT. They examined how the amount of protein made and the CpG-methylation marks on the primary human CRC tissues affected the cancer. The expression of CSF1R, CSF1, and IL34 in primary CRCs associated with a more mesenchymal-like subtype and poorer patient survival outcomes. It was determined that miR-34a and CSF1R had an inverse correlation, since miR-34a directly inhibits CSF1R. Additionally, p53 lessened the expression of CSF1R by raising miR-34a, whilst SNAIL amplified the expression by decreasing miR-34a both directly and indirectly in what can be seen as a feed-forward cycle. CSF1R is a receptor that was activated by a STAT3-mediated system to cause EMT, migration, colonization, and metastasis in CRC cells. The CpG-methylation of miR-34a increased the expression of CSF1R, which in turn generated a resistance to 5-FU in CRC cells. This process was demonstrated by an elevated expression of CSF1R from the tumor invasion edge to distant metastasis being associated with CpG-methylation of the miR-34a promoter in the primary CRC. This illustrates that the loss of miR-34a in tumor cells and its repression by CSF1R could have an effect on therapeutic and prognostic measures of colorectal cancer [188].

A specialized system of response causes the expression of antizyme; this protein functions to restrict the amount of polyamine found within a cell. When the quantity of cellular polyamine grows, the translation of antizyme mRNA transitions to a frame shift that increases the formation of a fully operational antizyme protein [189]. AZ binds to the ODC monomer, which deactivates ODC and triggers its destruction by the 26 S proteasome when ubiquitin is not present. This reduces the absorption of PA, while also accelerating its excretion. OAZ1 has had its chemical reactions thoroughly investigated. OAZ2 and OAZ3 are shown to be involved in translation when there is PA-induced frame-shifting. All these AZs bind to and inhibit ODC, which results in less PA intake [190, 191]. Li et al. planned a study to determine if miR-34a contributes to the pathophysiology of CCa MDR [192]. This study has found that cancer cells become resistant to chemotherapy when miR-34a expression is decreased. Reduced levels of miR-34a in both clinical cancer samples and in vitro-produced multidrug-resistant cancer cells were found to correlate with increased resistance to chemotherapy. As a result, functional regulation of miR-34a/OAZ2 signaling was essential for the successful treatment of cancer with chemotherapy. Multidrug-resistant cancer cells become more resistant to chemotherapy when miR-34a expression is decreased. Multidrug-resistant cancer cells from patients who are resistant to oxaliplatin treatment and in vitro-produced multidrug-resistant cancer cells were all found to have decreased miR-34a expression. Reduced miR-34a expression in these cancer cells correlated with increased resistance to chemotherapy. Functionally, miR-34a inhibition increased oxaliplatin resistance in chemosensitive HCT-8 cells while ectopic exogenous miR-34a synthesis allowed the reexposure of multidrug-resistant HCT-8/OR cells to oxaliplatin therapy. The functional regulation of miR-34a/OAZ2 signaling was essential for the successful treatment of cancer using chemotherapy. Thus, the inhibition of miR-34a/OAZ2 signaling by chemotherapeutic drugs is key to the development of MDR in cancer cells. Ultimately, the progression of antiapoptotic pathways and ATP-binding cassette (ABC) transporters connected to MDR is greatly increased as a result of the inhibition of miR-34a/OAZ2 signaling by chemotherapeutic drugs. It was shown through this study that the correct cellular response to cancer chemotherapy depends on the miR-34a/OAZ2 cascade [192].

In many cancers, PDGFRA is overexpressed and plays a crucial role in encouraging cell proliferation and distant metastasis [193–197].

**Fig. 3:**
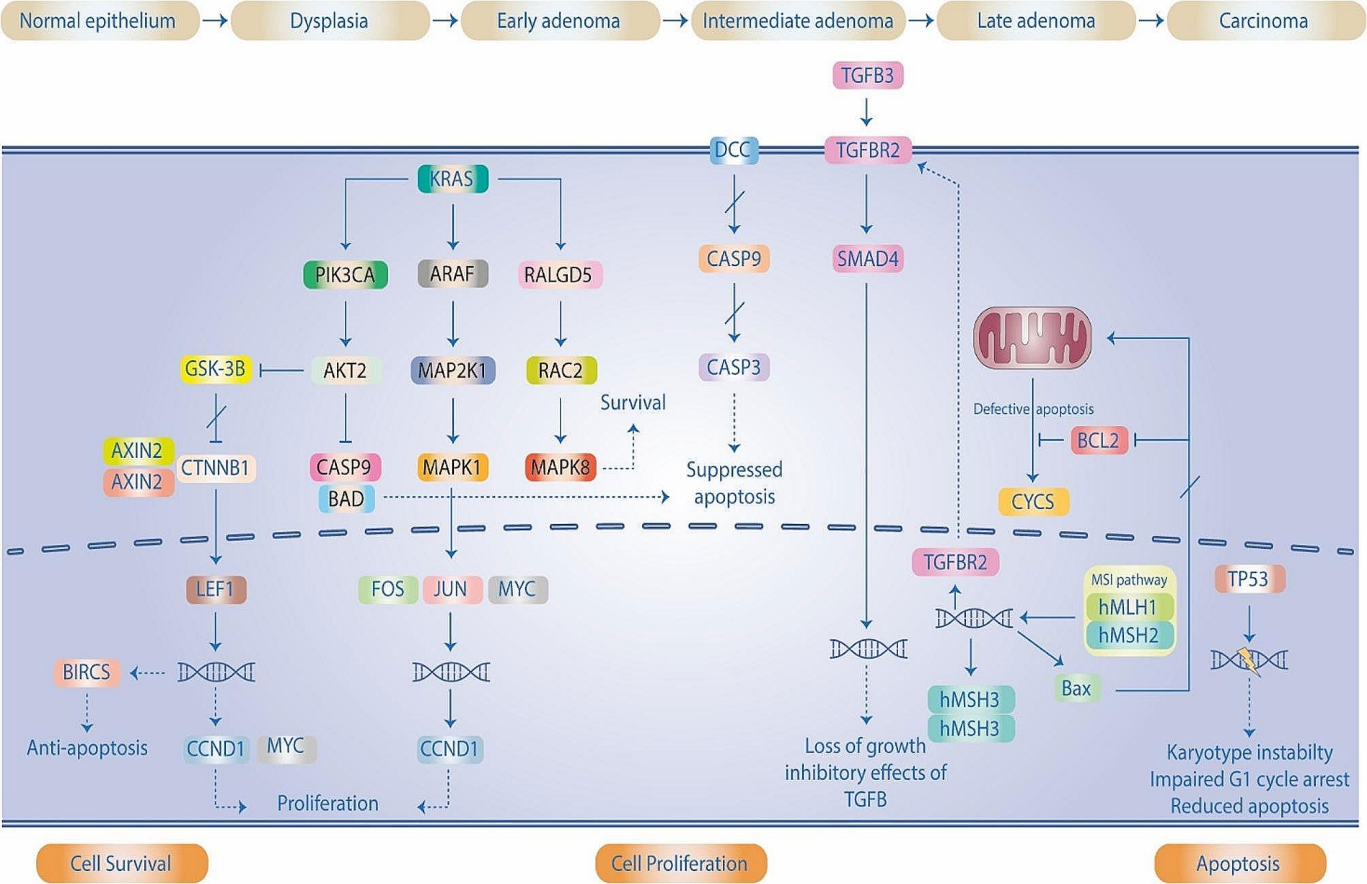
miR-34a has an effect on the genes associated with colorectal cancer, which have been highlighted in orange. This figure adapted from [199]

Li et al. explored the impact of miR-34a on the growth and propagation of colon cancer (Figure 3) [198]. Analysis of miR-34a expression began with studies indicating that levels of this microRNA were significantly lower in colon cancer tissues and cell lines than normal. Subsequent research then indicated that miR-34a had the capacity to induce G1 phase arrest, reduce the growth rate of colon cancer cells, restrict metastasis, and block epithelial mesenchymal transition. To further investigate these effects, bioinformatic and luciferase experiments went on to point to Platelet-derived growth factor receptor alpha (PDGFRA) as a direct target for miR-34a. Forwarding tests, involving Western blotting and quantitative reverse-transcription polymerase chain reaction analysis provided further evidence that miR-34a was instrumental in cutting the expression of PDGFRA in colon cancer cells. Functional assays verified that miR-34a exerted an inhibitory action on colon cancer by targeting PDGFRA. Taken together, this evidence implies that miR-34a has a suppressive effect on colon cancer through the regulation of PDGFRA [198]. Table 3 presents an overview of research conducted looking into the relationship between miR-34 and Colorectal Cancer (CRC).

**Table 3:** Various studies on miR-34 and CRC

Type of miR-34	Expression	Target (s)	Type of model	Tumor cell line	Citation
miR-34	Up	-	In vitro human	HT29	[200]
miR-34	down	Notch-1	Human In vitro	SW620, HCT116P53−/−, HCT116 CR	[201]
miR-34	Down	Survivin, MET	In vitro Human In vivo	HT-29, RKO, RKO-E6, HCT116 p53+/+ HCT116 p53-/-	[202]
miR-34	Up	-	Human	-	[19]
miR-34a	Down	CSF1R, SNAIL, ZNF281, IL6R, INH3, PAI-1, TPD52, AXL, PDGFR, c-Met, c-Kit, ZNF281, CD44	Human In vitro	HCT15, RKO, DLD1	[188]
miR-34a	Down	OAZ2	Human, In vitro, in vivo	HCT-8, HCT-116, SW-480	[192]
miR-34a	Down	-	Human In vitro	FHC, LOVO, SW480, SW620, HCT116, HT29	[203]
miR-34a	Down	PDGFRA	Human In vitro	HT‑29, SW620, Lovo, Colo205	[198]
miR-34a	down	SGPP1	Human In vitro In vivo	(ATCC® CCL‑228™, SW480; and ATCC® CCL‑229, LOVO	[204]
miR-34a	Down	Fra-1	In vitro In vivo	HEK293T, RKO, HCT116, HCT116 p53-/-	[205]
miR-34a	Down	-	Human	-	[206]
miR-34a	Up	Notch1, WNT1 ,CD44,c-Myc	In vitro In vivo	HCT-116, DLD-1	[207]
miR-34a	Down	E2F1	Human In vitro	Hct-116, Sw-480	[208]
miR-34a	down	Notch , Wnt	Human In vitro	Colo205, SW480, HT29, SW620, LS174T, DLD1, Caco-2	[209]
miR-34a	Up	-	human	-	[210]
miR-34a	down	IL-6R, IL-23R, CCL22, IL-17RD	human In vivo	-	[211]
miR-34a	Down	WNT1,WNT	Human In vitro In vivo	Lovo, SW480, HCT116, HT29, NCM460	[212]
miR-34a	down	HOTAIR	Human In vitro	HT29, HCT8, HCT-116, FHC	[213]
miR-34a	down	Notch1	Human In vivo In vitro	CCSCs	[214]
miR-34a	down	SIRT1, c-Myc, NOTCH1, MMP1, BCL2, KLF4	Human In vitro In vivo	HCT116, HCT116 p53− / −	[125]
miR-34a	down	SIRT1	Human In vitro In vivo	SW480, CaCO2, RKO, HCT8, HCT116, SW620, FHC	[215]
miR-34a	down	Numb, Notch1	In vivo In vitro	CCSCs	[216]
miR-34a	down	-	Human	-	[217]
miR-34a	down	SYT1	Human In vitro	SW620, SW480	[173]
miR-34a	down	c-Met, Snail, β-catenin	Human	-	[218]
miR-34a	Up	Bcl‑2, Notch1	In vitro In vivo	HCT116, LoVo, SW480, SW620, HT‑29, DLD‑1	[219]
miR-34a	Down	-	In vitro	DLD-1	[220]

## MircroRNA-34 and esophageal cancer

The E2F transcription factor 5 (E2F5) has an oncogenic function in a number of cancers, suggesting that E2f family transcription factors may be an essential downstream route [221].

Jiang et al.’s research aims to investigate miR-34a and E2F5’s inhibitory impact on ESCC [222]. In order to ascertain its therapeutic value, the researchers examined the miR-34a expression in various embryonic stem cell-related cancer (ESCC) cell lines and patient malignancies. To study its function in proliferation, apoptosis and migration, RNA mimics and inhibitors were employed and gain- and loss-of-function experiments on E2F5 were conducted. It was found that ESCC inherently expressed low levels of miR-34a. Yet, it had the capability to impede cancer cell proliferation, migration and was pro-apoptotic. Furthermore, researchers confirmed that miR-34a specifically attaches to E2F5 resulting in increased proliferation, mobility and survival of tumor cells. This is in accordance with the idea that miR-34a can regulate the cell-cycle regulator E2F5, which may be of great importance in comprehending the anti-cancer properties of miR-34a [222].

FNDC3B is a big region that may be found on chromosome 3 (q26.31) (360 kb). The fibronectin family includes FNDC3B [223] has a biological role that is yet poorly understood. An increasing total of oncogenic drivers have been uncovered in the heightened region of 3q amplicon, with FNDC3B being the most recently identified crucial oncogenic driver gene [224]. MMP-2 and MMP-9 of the MMP family, often referred to as gelatinases, are essential to the process of cancer invasion and progression due to their ability to degrade the type-IV collagen making up the basement membrane. At the onset of tumor invasion, these two particular MMPs become of great significance. As the tumors grow bigger, they generate chemicals that could alter the local microenvironment and consequently, change the way the tumor behaves [225].

Yang et al. investigated how miR-34 controls the growth of ESCC [17]. To validate the presence of miR-34a in ESCC tissue samples, the reverse transcription polymerase chain reaction was employed. Transwell assays and a wound healing test were conducted to evaluate how miR-34a affects ESCC cell line migration and invasion. To further study its influence, Western blotting and luciferase reporter assays were implemented. Quantitative polymerase chain reaction revealed that ESCC tissues featured far less miR-34a than neighboring normal tissues. Additionally, overexpression of miR-34a brought a marked decrease of numerous proteins, including MMP-2, MMP-9, and FNDC3B coding and 3’-untranslated sections, in ESCC cell migration and invasion. These outcomes indicate that microRNA 34a is able to impede cell migration and invasion in ESCC by lessening the expression of MMP-2, MMP-9, and FNDC3B [17].

A novel gene subfamily within the PLC family encodes the enzyme phospholipase C epitope 1 (PLCE1) [226]. To produce 3-phosphoinositide (IP3) and diacylglycerol (DAG), phospholipase C, gamma 1 (PLCE1) hydrolyzes membrane phosphatidyl inositol 4, 5 diphosphate (PIP2), which in turn regulates metabolism, cell growth, and distinctions [227]. The significance of PLCE1 is shown with its role in a variety of important human malignant tumors, including skin cancer [228, 229], bladder [230, 231], colorectal [232, 233], neck and head [234, 235] and ESCC cancers [236, 237]. Nevertheless, PLCE1 acts as an oncogene in endometrial, urothelial, and bladder cancers while acting as a tumour suppressor in colorectal cancers (ESCCs).

Phospholipase C elipson 1 (PLCE1), a gene linked to disease development, was specifically demonstrated by Cui et al. to be expressed at higher levels in esophageal carcinomas [238]. This research demonstrated the relevance of miRNAs in managing the expression of their respective genes. They observed that tissue containing esophageal squamous cell carcinoma demonstrated a dramatic decrease in the amount of miR-34a expressed [239]. In addition, experiments were conducted on cell lines of ESCC where miR-34a was either activated or decreased to find out its role. Results indicated that PLCE1 was a direct, functional target of miR-34a; its action as a tumor suppressor was further validated since it reduced the growth rate, hampered the migration of cells and reversed the EMT characteristics of ESCC cell lines, increasing the cells’ sensitivity to apoptosis. Further research has been conducted on the relationship between PLCE1 and MiR-34a in ESCC tumors, which has increased our understanding of the tumorigenicity in both in vivo and in vitro settings. Their studies have revealed that miR-34a functions to suppress the expression of PLCE1 and as a result, inhibit the development of cancer. Because of these new findings, the miR-34a/PLCE1 axis has become a potential target for the treatment of ESCC, in addition to providing detailed insights into the molecular mechanism of ESCC metastasis [239].

MiR-34a has been shown to target cyclin D1, CDK4, CDK6, E2F3, Bcl-2, SIRT1, cyclin E2, and MYCN, enhancing G0/G1 arrest and apoptosis [14, 240, 241].

Shi et al. utilized qRT-PCR to analyze the performance and significance of miR-34a in esophageal cancer. For their research, they collected samples from both human esophageal cancer tissue and adjacent esophageal tissue [242]. . In order to examine the impact of miR-34a on human esophageal cancer cells, four separate assays were applied: the CCK8 assay, flow cytometry, in vitro migration tests, and MSP assays. It was established that miR-34a was expressed at lower levels in human esophageal cancer tissues compared to other types of cells. After the ectopic expression of miR-34a in esophageal cancer cells, cell growth and migration were suppressed. Western blotting experiments revealed that miR-34a caused levels of cyclin D1 and CDK6 to decrease. Although p53 expression was not related to miR-34a, it was demonstrated that 5-AZA-dC reduced miR-34a’s DNA methylation making it a possible mechanism of miR-34a’s inhibition of human esophageal cancer [242]. Table 4 presents various research projects that have investigated the connection between miR-34 and esophageal cancer.

**Table 4:** Various studies on miR-34 and esophageal cancer

Type of miR-34	Expression	Target (s)	Type of model	Tumor cell line	Citation
miR-34a	Down	E2F5	Human In vitro	Eca109, KYSE150, EC9706, TE-1, and HEEC	[222]
miR-34a	Down	MMP-2, MMP-9, and FNDC3B	Human In vitro	EC9706, TE-1	[17]
miR-34a	Down	cyclin D1, CDK6	Human In vitro In vivo	ECA109, TE1	[242]
miR-34a	Down	-	Human In vitro	EC1, KYSE190	[243]
miR-34a	down	-	Human	-	[244]
miR-34a	up	-	Human	-	[245]
miR-34a	down	-	In vitro	TE4, TE10, TE11, TE15	[246]
miR-34a	down	-	In vitro In vivo	KYSE-450, KYSE‐410, ECa-109	[247]
miR-34a	down	-	Human	-	[248]
miR-34a	down	-	In vitro	EC109, KYSE450	[249]
miR-34a	down	c-Met, cyclin D1	Human In vitro	TE-1, TE-3, TE-7, TE-8, HCE-4, HCE-7, SKGT-4, SKGT-5, Seg-1, Bic-1	[250]
miR-34a	down	PLCE1	Human In vitro In vivo	Eca109, KYSE150, EC9706, TE-1	[239]
miR-34a	down	-	In vitro	TE8	[251]
miR-34b/c	down	-	Human	-	[105]

## Exosomal microRNA-34 and gastrointestinal cancers

Exosomes are nanoparticles that range in size from 30 to 150 nm and have been found in a number of physiological fluids [252]. Exosomes have been found to be effective carriers for transporting miRNA into cells in a recent research. Exosomes are easier to enter cells than other conventional gene carriers because their phospholipid structure is similar to that of the cell membrane [253]. Exosomes are endogenous vesicles that often do not provoke an immune response when they reach the human circulatory system [254]. Multiple studies published during the last decade demonstrate that exosomes may efficiently transport siRNA, DNA, and proteins to their intended recipient cells [255].

The study carried out by Zuo et al. explored the production of miR-34a, which was covered with exosomes, in relation to its anti-cancer effects in pancreatic cancer [256]. Using confocal microscopy and flow cytometry, exomiR-34a’s transfection efficiency was evaluated after it was synthesized using an ultrasonic technique. Real-time quantitative PCR was utilized to assess miR-34a and the gene it inhibits, Bcl-2 (qRT-PCR). To confirm the effects of exomiR-34a on pancreatic cancer, the development of the cells was monitored with the MTT assay. In addition, Western blotting and Annexin-V/PI double staining were performed to measure the apoptosis of the cells. To further investigate the anti-cancer properties of exomiR-34a, a xenograft model in naked mice containing human Panc28 pancreatic cancer cells can be utilized. The capacity to cross the cell membrane and modify the expression of genes related to apoptosis make ExomiR-34a a powerful tool in the fight against pancreatic cancer. Inhibition of Bcl-2 expression was found to cause the death of cancerous cells as well as slow the proliferation of Panc28 cells. An in vivo study using xenograft naked mice further showed that tumor creation could be greatly delayed when treated with this exomiR. The results of both in vivo and in vitro studies suggest that exomiR-34a may be an effective means of stopping the spread of pancreatic cancer. Therefore, researchers came to the conclusion that ExomiR-34a may be a highly impactful therapeutic approach for human pancreatic cancer [256].

Exosomes are desirable nano-carriers because of their biocompatibility, lack of immunogenicity, lack of toxicity, permeability through biological barriers, and stability in the bloodstream [257]. It was also shown that the exosomal payloads could differ based on the biological source of the exosomes and the local micro - environment. For instance, texosomes, a kind of tumor-derived exosomes (TEX), may convey various signaling molecules to neighboring cells, which can then impact cellular functions and the pathogenesis of cancer [258]. Hosseini et al. studied the preventive and progressive influences of the freshly devised nano-carrier composite by encapsulating miR-34a into the cancer of the colon cell line CT-26 using exosomes obtained from tumors (TEXs) [259]. Sample cells CT-26 lacking nutrients were incubated in a buffer containing CaCl2, which had been loaded with miR-34a-containing exosomes. The CT-26 cells were then differentially exposed to varying doses of either TEXs or TEX-miR-34a, and their viability was observed. The survival rate and rate of movement of CT-26 cells exposed to TEX-miR-34a and TEX were looked into. Furthermore, the expression levels of IL-6R, STAT3, PD-L1, and VEGF-A, key molecules associated with tumor formation, were examined in the treated CT-26 cells to study the effects on tumor proliferation. The results indicated that with TEX-miR-34a, the rate of apoptosis in CT-26 cells amplified in accordance with the quantity of the treatment. Furthermore, the levels of IL-6R, STAT3, PD-L1, and VEGF-A in the treated CT-26 cells were drastically decreased. The TEX-miR-34a treated cells had a weaker capacity to migrate than the untreated ones. There was a noticeable decrease in the proliferation and migration of tumor cells once they were exposed to TEXs enhanced with miR-34a. miR-34a entry into tumor cells via exosomes released by starved CT-26 cells was effectively possible, which regulates the genes involved in tumorogenesis. Despite there being no direct effect on cancer cells, TEXs have the potential to act as an adjuvant in the treatment of CRC [259]. Fibroblasts associated with cancer are a crucial element of the milieu around a tumour and are necessary for the growth and propagation of the cancer [260–262]. It has been seen that CAF-released exosomes are a vital molecule-carrier for intercellular communication [263–265]. The goal of Shi et al.‘s study was to determine how GC synthesis and associated operations are influenced by CAFs and associated exosomes [266]. The study showed that miRNA-34 concentrations were lower in GCFs and GC cell lines. When scientists increased miRNA-34’s levels in the GC lines, the ability of the cells to spread, move, and proliferate decreased. What’s more, when GCFs with higher levels of miRNA-34 were incorporated into the GC cells, aggressive growth was inhibited. Furthermore, supporting evidence for the GCFs’ roles as anti-tumor agents was found through both in vivo and in vitro trials; taking up GCF-produced exosomes via the GC cells. The exosomal miRNA-34 diminished tumor growth in vivo and impeded the GC cells’ invasion and proliferation in vitro. Finally, examination found 16 genes that had the possibility of being targeted by miRNA-34 as downstream targets. When taken together, the capacity of exosomal miRNA-34 produced by GCFs to act as a therapeutic agent for gastrointestinal cancers was revealed [266]. Table 5 lists various studies on exosomal miR-34 and GI cancers.

**Table 5:** Various studies on exosomal miR-34 and GI cancers

Cargo	Target (s)	Type of Model	Tumor cell line	Citation
miR-34	AR, CCL22, CCND1, CCNE2, CDK4, CDK6, c-Met, E2F3, E2F5, HMGA2, LETK3, MTA2, N-Myc, PAR2, SFRS2, ,SIRT1	In vitro In vivo	AGS, AZ52, MKN1, NUGC3, GCFs, CFs	[266]
miR-34	-	In vivo	-	[142]
miR-34a	-	human	-	[267]
miR-34a	Bcl-2	In vitro In vivo	HEK293, HPDE6-C7, Miapaca-2, Panc28	[256]
miR-34a	IL-6R, STAT3, VEGF-A, and PD-L1	In vitro	CT-26	[259]

## Conclusion

The prognosis of cancer is poor due to the spread of cancer cells. The miR-34 family has been studied for its potential to act as a tumor suppressor, slowing the malignancy’s growth and carcinogenesis. MiR-34a has been the focus of countless investigations and has been found to possess a tumor-suppressing effect. Issues such as the relatively quick degradation of miRNA and an immune response to treatments featuring the miRNA present roadblocks to its utilization. The need for nano-carriers has been established, but even then, their toxicity and the possibility for undesired side effects could prove fatal. Despite these obstacles, miR-34a remains a promising choice for cancer treatment, and the miR-34 family as a whole should continue to be further explored for its potential applications in oncotherapy.

## Data Availability

Not applicable.
